# A 1915 Code of Dental Ethics

**Published:** 1915-01

**Authors:** Herbert E. Dennett


### A 1915 CODE OF DENTAL ETHICS.

BY HERBERT E. DENNETT, D.D.S.

Looking back over fifty years of an ethical dental practice, I draw the following conclusions for today:

Dental Ethics is dual, as it concerns both the profession and the laity.

In time of need the ethical dentist is the patient's best friend.

The ethical patient should be the dentist's best friend.

Ethical dentists are entitled to consultation fees.

Unethical dentists are liable to malpractice suits.

Ethical patients point to all "free bait" with suspicion.

Unethical patients are largely responsible for the existence of unethical dentists, who have robbed millions of mouths of teeth which might have been made serviceable and substituted for them the present day cheap apology, loaded with slow poisons.

Ethical dentists recommend that ounce of prevention which forestalls the pound of cure.

The ethical patient or dentist, will so far as possible, present a sanitary mouth and breath to whom it may concern.

An offensive breath is indicative of decaying morals.

The ethical dental operator will not go to the mouth with unsterilized hands or instruments.

Fixed bridges obstruct mouth hygiene, form food traps which become incubators and brooders of disease, inexhaustible generators of offensive breath and make their owners unwelcome guests to respectable society.

Degrading habits curtail efficiency, induce disease and disqualify the candidate for ethical dental practice.

Conservation is the keynote to dental ethics.

The heroic dentist is unethical—a natural coward and mainly responsible for the fear of the dental chair—witness teethless mouths.

The dental mallet is a worse than useless instrument of torture.

The dental machinery in common use should not be used except with uncommon care.

One careless jab of steel into the live pulp means instant needless torture, usually followed by its death and other serious complications.

To remove a tooth which can be "saved"—made serviceable—destroy the vitality—"kill the nerve"—remove sound dental tissue for convenience of filling or fill with base metals singly or in combination is the antithesis of conservation. The least hurt or injured tooth is the one best treated. Any bungler can inflict pain which the skillful operator avoids.

Alveolar pyorrhea can not be cured except by removing the cause and right feeding.

To stop the discharge of pus without removing the cause is to force it to find a new outlet, become piled up in the system or cause blood poison.

That a "clean tooth never decays" is a too often repeated falsism.

That some are worse than others is the best that can be said of dentifrices.

Children need teeth as soon and as long as they need solid food.

To wait for public school inspection is gross negligence on the part of parents and guardians.

It is the duty of the ethical dentist to educate the laity to the true cause of dental disorder, its prevention and cure.

Dental disorder and associate ills may be discounted by timely right feeding.

Right feeding spells conservation from the ground up. It includes perfect ensalivation. Perfect ensalivation is implied absence of "washing down."

Wholesome food for man and animals can be harvested only from the soil rightly fed.

Cremation of filth and scientific combination of stones from Nature's exhaustless storehouse will put the farm pests out of commission, cause the earth to bloom like a rose and produce abundant harvest.

From Stone to Plant, from Plant to Animal, from Animal to Man, from Man to God is a great cabalistic truism.

About fifty-one grains of organized stone is daily needed to keep the average human body in repair.

Boltless milling of grain, waterless cooking of vegetables and the elimination of fermentation each contribute their proper quota to conservation.

The sugar-glucose-candy craze threatens the extinction of the human race.

Will the many readers kindly forward their protests or approval of the foregoing and oblige, to Dr. H. E. Dennett, 1325 Commonwealth Ave., Boston.

